# Case report: Clinicopathological characteristics of *SASH1* mutation-related dyschromatosis: a rethinking of the classification of dyschromatosis

**DOI:** 10.3389/fgene.2025.1414129

**Published:** 2025-03-06

**Authors:** Tingmei Wang, Dong Li, Yunhua Deng

**Affiliations:** Department of Dermatology, Tongji Hospital, Tongji Medical College, Huazhong University of Science and Technology, Wuhan, China

**Keywords:** *SASH1*, dyschromatosis, dyschromatosis universalis hereditaria, multiple lentigines, *ABCB6*

## Abstract

Dyschromatosis, a group of pigmentary dermatoses, accompany both hyper- and hypo-pigmentation, including dyschromatosis symmetrica hereditaria (DSH), dyschromatosis universalis hereditaria (DUH), and familial progressive hyper- and hypo-pigmentation (FPHH). A peculiar phenotype of dyschromatosis presented as multiple lentigines and hypopigmentation with various sizes and shapes was found to be associated with *SASH1* mutations and has recently been reported frequently. The current study evaluated the clinical manifestation, pathological pattern, and genetic basis of dyschromatosis in a five-generation family. This research also presents a case study of a sporadic patient with dyschromatosis caused by *SASH1* mutations and shows different clinicopathological characteristics form DSH, DUH and FPHH. SASH1 (SAM and SH3 Domain Containing 1) gene, located on chromosome 6q24.3, encodes a tumor suppressor protein involved in cell signaling, migration, and adhesion. Additionally, the *SASH1* mutations could also lead to another pigmentary phenotype: multiple lentigines. High consistency in clinicopathological features and genetic basis in these two *SASH1*-related pigmentary disorders suggests that *SASH1* mutations cause multiple lentigines and dyschromatosis which might belong to a disease spectrum. Overall, it is expected the current study results could help enhance a more comprehensive understanding of *SASH1*-related pigmentary dermatoses.

## Introduction

The term “dyschromatosis” refers to a group of pigmentary disorders that present with both hyper- and hypo-pigmentation. Three known subtypes of this disease are dyschromatosis symmetrica hereditaria (DSH), dyschromatosis universalis hereditaria (DUH), and familial progressive hyper- and hypopigmentation (FPHH); they result from autosomal dominant inheritance without systemic involvement; DSH, DUH, and FPHH are caused by adenosine deaminase acting on RNA1 gene (*ADAR1*) (Miyamura et al., 2003), ATP binding cassette subfamily B member 6 gene (*ABCB6*) (Zhang et al., 2013), and KIT ligand gene (*KITLG*) (Amyere et al., 2011), respectively. Recently, several studies (Zhou et al., 2013; Zhong et al., 2019; Wu et al., 2020; Liu et al., 2021; Cao et al., 2021; Yang et al., 2023) have documented a distinct group of dyschromatosis in an autosomal dominant inherence pattern caused by mutations of *SAM* (sterile alpha motif) and *SH3* (Src homology domain 3) domain-containing protein 1 gene (*SASH1*). The dyschromatosis caused by *SASH1* mutations is sometimes considered to be a subtype of DUH. However, the objective of the present study is to focus on the clinical manifestation and genetic basis behind *SASH1-*related dyschromatosis despite the lack of adequate pathological analysis. In this study, the clinicopathologic features of the dyschromatosis caused by *SASH1* mutations are analyzed to gain a better understanding of this disorder and make a clearer classification of dyschromatosis without systemic involvement.

## Case presentation

### Pedigree

**FIGURE 1:**
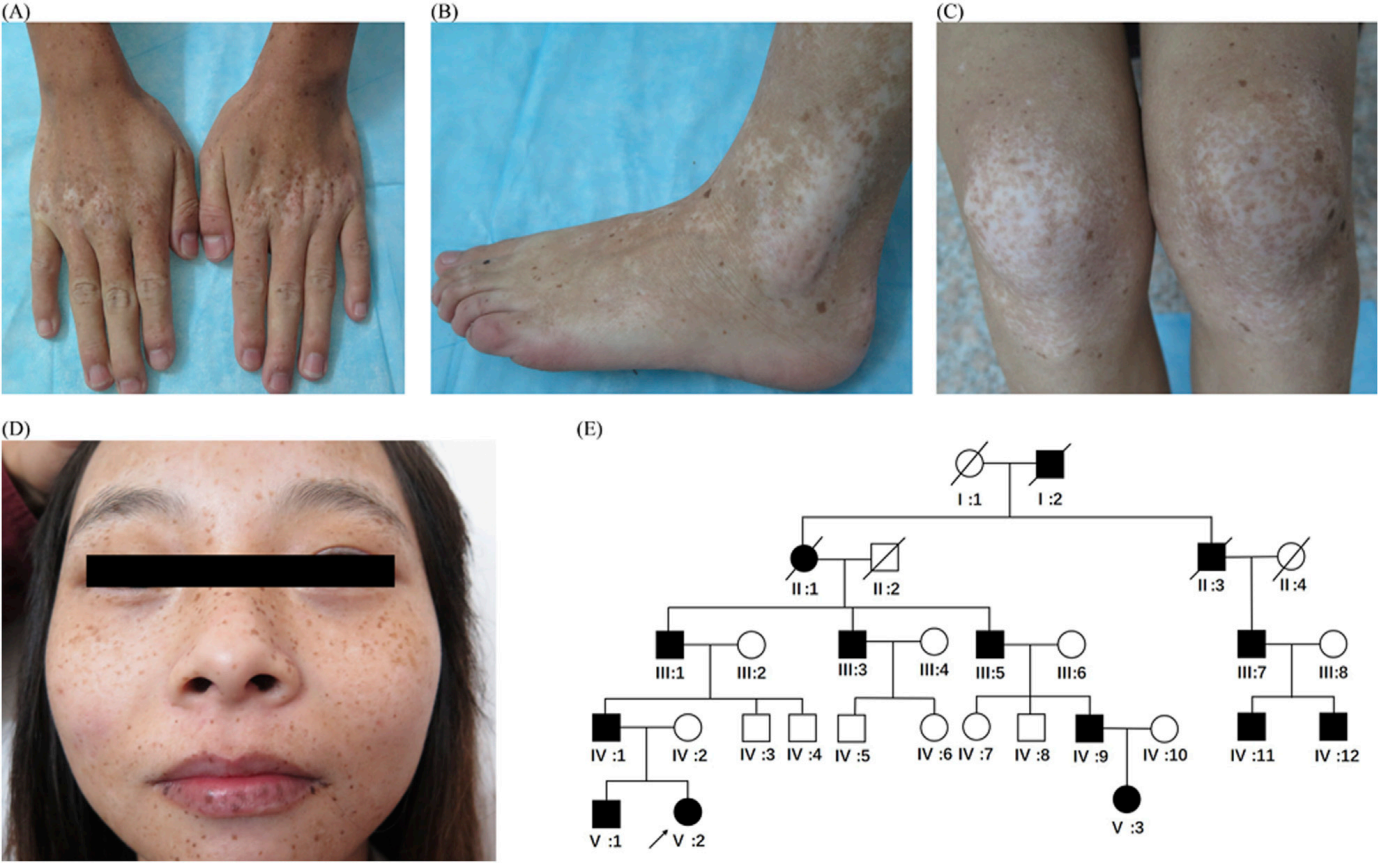
Clinical features and pedigree **(A–D)** Clinical features of the proband (V:2). Multiple hypopigmented macules or patches mixed with lentigo-like macules on the dorsa of the hands **(A)**, feet **(B)** extension of knee joints, **(C)** and face and lips **(D)**. **(E)** Pedigree chart. Black symbols represent affected individuals, and open symbols represent unaffected individuals. Circles and squares indicate women and men, respectively. The arrow indicates the proband.

The proband, a 20-year-old woman from central China, presents with cutaneous hyper- and hypopigmentation lasting 19 years. The condition started as multiple lentigo-like lesions on her face when the patient was 1 year old. Later on, some hypopigmented macules developed on the dorsa of interphalangeal and metacarpophalangeal joints of her hands. Over time, these macules extended to her feet, hands, trunk, face, and other limbs. Slight canities appeared in her hair at the age of 19. Her palms, soles, and nails were spared. She displayed normal intellectual ability and no other systemic or developmental defects. Dermatological examination revealed multiple ill-demarcated hypopigmented macules and patches mixed with well-circumscribed, lentigo-like hyperpigmentation on the dorsa of hands, feet, arms, legs, buttocks, trunk, neck, and face (Figures 1A–D). The most pronounced features of this proband were the symmetrical hypopigmentation on the joint prominences and the hyperpigmentation presented as multiple lentigines (Figures 1A–C).

**FIGURE 2:**
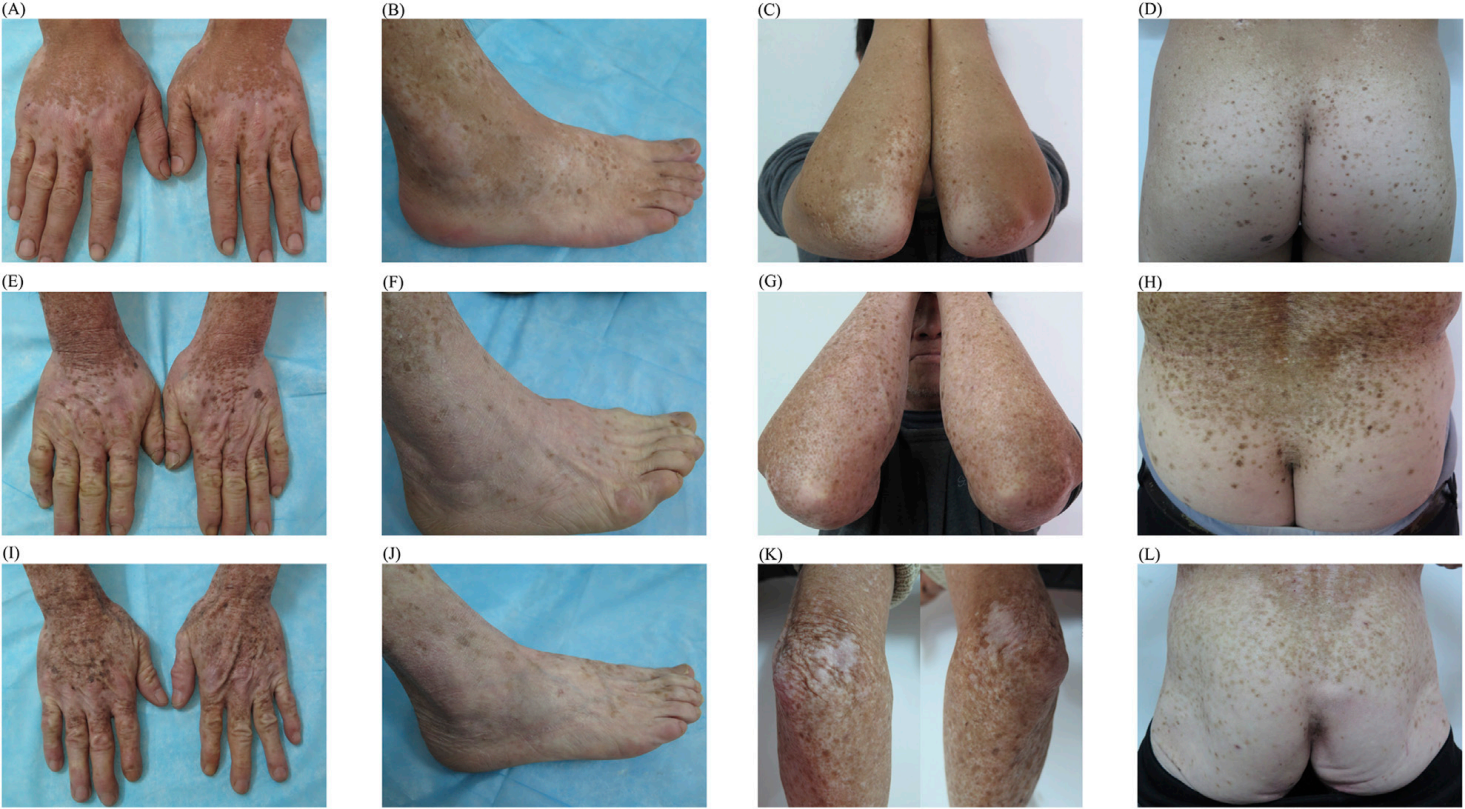
Typical manifestations of individuals of different ages within family **(A–D)** Diffuse hypopigmentation with hyperpigmented macules on the dorsa of the extremities, extensor surfaces of elbows, and buttocks of individual IV 9 **(E–H)** Similar manifestations on individual III 5 **(I–L)** Similar manifestations on individual III 3.

All living members of the patient’s pedigree underwent a thorough medical evaluation. An autosomal dominant pattern was observed (Figure 1E). Fourteen individuals (11 men and 3 women) were affected, and the 11 surviving affected members showed similar manifestations and clinical course. Hypopigmentation was apparent on the bilateral dorsa of their extremities (Figures 2A, B, E, F, I, J) and extensor surfaces of the knees and elbows (Figures 2C, G, K) of the individuals. Diffuse hypopigmentation with some hyperpigmented macules often developed on their buttocks during their thirties (Figures 2D, H, L). Canities usually began around age 20 and gradually worsened (Supplementary Figure S1).

### Sporadic patient

The sporadic patient studied here is a 24-year-old man from central China; he presented with cutaneous hyper-and hypopigmentation lasting 23 years. The clinical manifestations of this patient closely resembled those of the collected dyschromatosis family. His dermatological examination revealed multiple lentigo-like hyperpigmentation, and intermixed or confluent hypopigmented patches over the whole body. The hypopigmentation on the buttocks and lower limbs was diffuse and homogeneous (Supplementary Figure S2). Contrastively, his elder sister and both parents had no similar pigmentation defects.

### Molecular analysis

**FIGURE 3:**
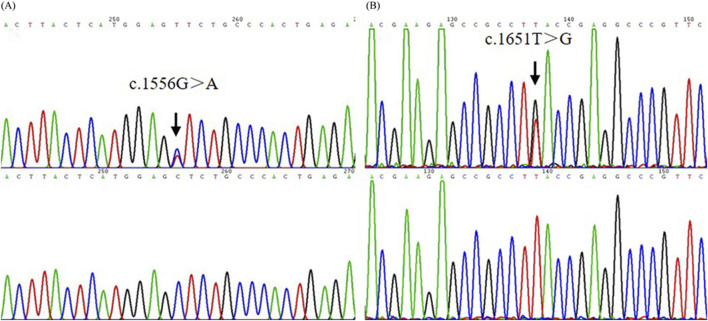
The mutation analysis of *SASH1* in the pedigree and sporadic patient. **(A)** A heterozygous missense mutation c.1556G>A (S519N) in *SASH1* was identified in the pedigree. **(B)** A heterozygous missense mutation c.1651T>G (Y551D) in *SASH1* was identified in an additional individual.

Pathogenic mutations of *ADAR1*, *ABCB6,* and *KITLG*, the respective causative genes for DSH, DUH, and FPHH, were screened using Sanger sequencing in the proband of dyschromatosis pedigree. No causative mutation was identified in *ADAR1*, *ABCB6,* or *KITLG* genes. Thereafter, the mutations of *SASH1* in this patient were screened, which resulted in discovery of missense mutation c.1556G>A p. Ser519Asn (S519N)(Figure 3A) in this dyschromatosis pedigree. S519N mutation was confirmed in the patients of this family, though it was absent in other unaffected individuals. This variant was co-segregated perfectly with the phenotype in this family.

The other missense mutation c.1651T>G p. Tyr551Asp (Y551D) in *SASH1* was detected in the sporadic patient of dyschromatosis and was not observed in his parents and elder sister (Figure 3B). Further direct DNA sequence analysis with a panel of 300 unaffected control individuals matched for the geographical location could not detect these two mutations.

### Pathological findings

**FIGURE 4:**
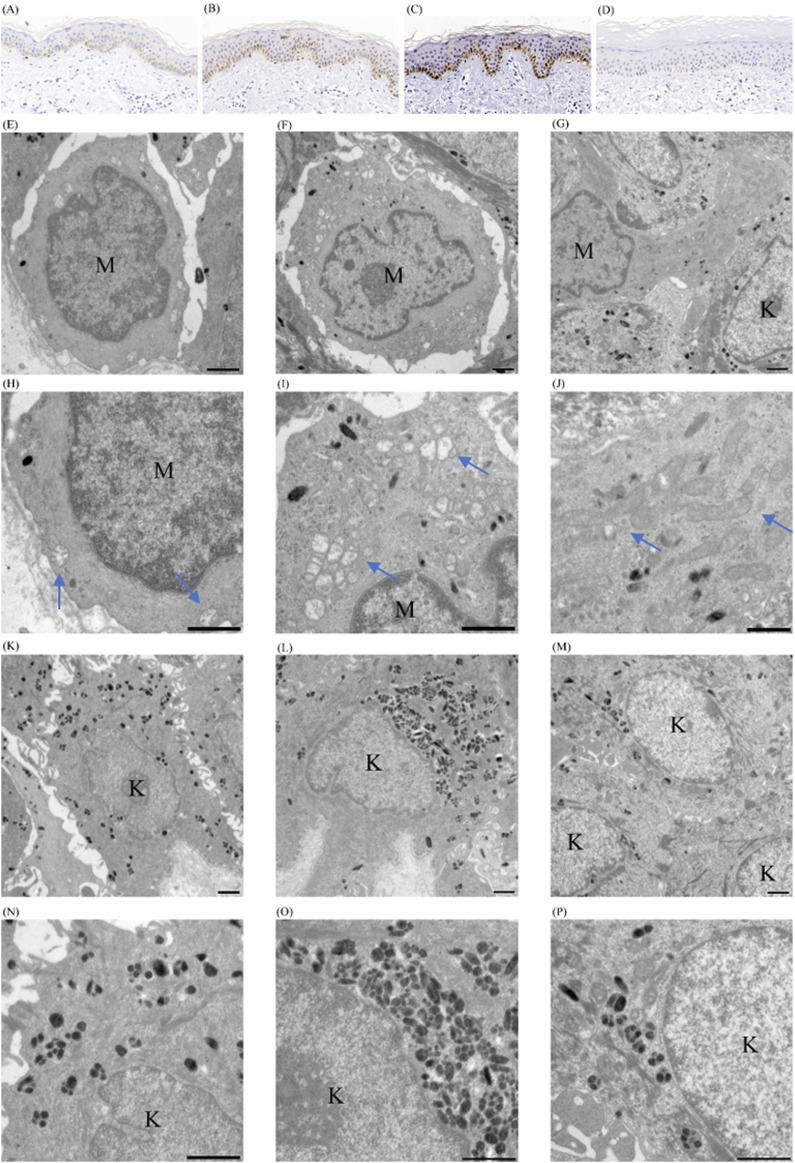
Pathological findings of cutaneous tissues of patient with *SASH1* mutation related dyschromatosis. **(A–D)** Immunohistochemical analyses on the cutaneous tissues. MITF immunohistochemical staining of normally **(A)**, hyper-**(B),** and hypo-**(C)** pigmented cutaneous tissues of patient with *SASH1* related dyschromatosis, and of cutaneous tissues of normally controls **(D)**, original magnification ×200 **(E–P)** Ultrastructural analyses on the cutaneous lesions. The melanocytes in normally pigmented areas showed smaller cell bodies and poor dendritic formation, loose connection with their surroundings and contained fewer melanosomes and mitochondria **(E)**. The melanocytes in hyperpigmented areas showed poor dendritic formation, loose connection with their surroundings, and contain moderate melanosomes and mitochondria **(F)**. The melanocyte in hypopigmented lesions was large in size with elongated dendrites and contained numerous melanosomes and mitochondria **(G)**. Fragmentation and swelling with obscure cristae and vacuolization of mitochondria were observed in both normal- and hyper-pigmented areas. The morphology abnormalities of mitochondria were significantly more serious in melanocytes from normally pigmented areas **(H, I)**. The mitochondria in hypopigmented lesions were normal in morphology and interconnected presenting as intracellular networks **(J)**. More and fewer melanosomes within keratinocytes of hyper- **(L)** and hypopigmented **(M)** than in normally pigmented skin **(K)**. The melanosomes were primarily distributed individually with a few non-membrane-bound melanosome complexes in normally pigmented keratinocytes **(N)**. The non-membrane-bound melanosome complexes predominated in hyperpigmented keratinocytes **(O)**. The membrane-bound melanosome complexes predominated in hypopigmented keratinocytes **(P)**. The bar indicates 1 μm; the arrow indicates mitochondria. (MC melanocyte; KC keratinocyte).

Microphthalmia-associated Transcription Factor (MITF) was used to examine if a change in the number of melanocytes participates in the occurrence and development of this dyschromatosis and to label the melanocytes in the specimens from the normally, hyper- and hypo-pigmented areas and from the skin of the normal control. The cells stained positively by the melanocyte marker MITF were counted under three different fields of ×200 magnification: 13.0 ± 0.7 (mean ± CD) in normally pigmented areas (Figure 4A), 10.3 ± 2.1 (mean ± CD) in hyperpigmented lesions (Figure 4B), and 5 ± 0.82 (mean ± CD) in the skin of unaffected controls (Figure 4D). However, only hyperpigmented lesions demonstrated a pronounced increase in melanin (Figure 4B), although both the normally pigmented and hyperpigmented skin showed increased melanocytes. Furthermore, only one MITF-positive cell was detected in any of the hypopigmented lesions on the specimen (Figure 4C).

At an ultrastructural level, the melanocytes in normally pigmented areas showed smaller cell bodies with poor dendritic formation and contained fewer melanosomes and mitochondria-related to energy generation and protein synthesis (Figures 4E, H). The poorly activated morphology of melanocytes in normally pigmented areas shows an absence of any obvious hyperpigmentation despite an increased number of melanocytes in this area. Fragmentation and swelling with obscure cristae and vacuolization were observed in the mitochondria of both normally and hyper-pigmented areas (Figures 4E, F, H, I) in the patient. However, the fragmentation and swelling of mitochondria were significantly more serious in melanocytes from normally pigmented skin (Figures 4E, F, H, I) which might be the reason for the scarce melanosomes and mitochondria in normally pigmented melanocytes. Contrastively, the remaining melanocytes in hypopigmented lesions were large in size with elongated dendrites and contained numerous melanosomes and mitochondria (Figures 4G, J). The hypopigmented lesions showed normal mitochondria in morphology and interconnected building intracellular networks (Figure 4J). The overactived state is pointed to melannocytes rahter than lesions might be associated with the functional compensatory enhancement of the remanent melanocytes. In addition, both melanocytes from normally pigmented areas and hyperpigmented lesions were loosely connected with their surroundings (Figures 4E, F).

A higher number of melanosomes were observed within keratinocytes from hyperpigmented lesions than normally pigmented areas (Figures 4K, L), and fewer melanosomes were found within keratinocytes in hypopigmented lesions (Figure 4M). The distribution of melanosomes within keratinocytes from different sources was irregular. The melanosomes were predominantly distributed individually with a few non-membrane-bound melanosome complexes documented in normally pigmented keratinocytes (Figure 4N). The non-membrane-bound melanosome complexes were predominant in hyperpigmented keratinocytes (Figure 4O), whereas membrane-bound melanosome complexes predominated in hypopigmented keratinocytes (Figure 4P). This distribution pattern supports previously reported distribution of melanosomes in keratinocytes from different skin types (Hurbain et al., 2018).

## Discussion

In the form of dyschromatosis related to *SASH1* mutations, the lentigo-like macules and hypopigmented macules or patches started on the face or on the dorsa of interphalangeal and metacarpophalangeal joints of hands in infancy or early childhood and extended progressively onto the body with age. The most prominent feature of *SASH1*-related dyschromatosis is multiple lentigo-like macules coexisting with variable and symmetrical hypopigmentation which was prominent on the joint protuberances. The hypopigmented patches could progress to be diffuse and homogeneous. To our knowledge, premature canities were first observed in the present study. No other systemic or developmental defects appeared (Zhou et al., 2013; Zhong et al., 2019; Wu et al., 2020; Liu et al., 2021; Cao et al., 2021; Yang et al., 2023).

DSH, DUH, and FPHH are documented dyschromatosis subtypes of autosomal dominant inheritance without systemic involvement. A majority of *SASH1*-related dyschromatosis was reported as a subtype of DUH. *ABCB6* gene was first reported as the disease-causing gene of DUH in a five-generation Chinese family in 2013 (Zhang et al., 2013); other studies (Cui et al., 2013; Liu et al., 2014)also corroborated this finding subsequently. However, the *SASH1*-related dyschromatosis presented as a distinct clinical phenotype different from that in patients with DUH. DUH, a generalized dyschromatosis, is characterized by similar-sized small hyper- and hypo-pigmented macules intermixed in a reticular or mottled pattern (Zhang et al., 2013; Cui et al., 2013; Liu et al., 2014). Though, the multiple lentigo-like macules and the symmetrical hypopigmented patches, especially on the joint prominences, are the clinical characteristics of *SASH1*-related dyschromatosis but they are not commonly seen in patients with DUH (Zhang et al., 2013; Cui et al., 2013; Liu et al., 2014). Moreover, patients with DUH lack diffuse or homogenous hypopigmentation (Zhang et al., 2013; Cui et al., 2013; Liu et al., 2014).

To elucidate whether *SASH1*-related dyschromatosis is a subtype of DUH, we analyzed the pathological manifestations of *SASH1*-related dyschromatosis. The pathological results also indicated that *SASH1*-related dyschromatosis and DUH may be different entities. In the current study, changes in the number of melanocytes were found to be involved in *SASH1*-related dyschromatosis: the number of melanocytes showed approximately a 2-fold increase in both normally pigmented and hyperpigmented skin than normal controls and a remarkable decrease in hypopigmented skin. Also, the mitochondrial abnormalities within melanocytes were another pathological feature of *SASH1*-related dyschromatosis. The more serious fragmentation and vacuolization of mitochondria within normally pigmented melanocytes might be related to the decreased number of mitochondria, and might ultimately influence the melanogenesis and transport of melanin resulting in no obvious pigment deposit despite the increased number of melanocytes in normally pigmented skin of *SASH1* related dyschromatosis. DUH is not a pigmentary disorder related to the changes in melanocyte numbers (Cui et al., 2013). In addition, the abnormalities of mitochondria are not found in the melanocytes of the skin of patients with DUH (Gupta et al., 2015). Therefore, the clinicopathological features do not support the idea that DUH and *SASH1*-related dyschromatosis are identical entities.

DSH, caused by mutations in the *ADAR1* gene (Miyamura et al., 2003), is characterized by a mixture of hyperpigmented and hypopigmented macules distributed on the dorsal aspects of the hands and feet (Miyamura et al., 2003; Kondo et al., 2008). There are some similarities in their early age of onset, intermixture of hyper- and hypopigmentation on the dorsa of hands and feet, freckle-like lesions on the face, and histological feature of the loss of melanocytes in hypopigmented lesions. However, the confluent patches of hypopigmentation, even the diffuse hypopigmentation are not commonly observed in patients with DSH. Furthermore, the generalized distribution of lesions differed from DSH in the present study which is a localized pigmentary dermatosis. Although the hypopigmentation is caused by the missing melanocytes in the two cases, the current case study showed no degenerative melanocyte morphology in hypopigmented lesions, thereby indicating that different mechanisms might underlie the formation of hypopigmentation in DSH and the *SASH1*-related dyschromatosis (Kondo et al., 2008).

Clinical signs of FPHH consist of progressive diffuse hyperpigmented lesions, multiple café-au-lait macules, intermingled with scattered hypopigmented maculae, and lentigines (Amyere et al., 2011; Zanardo et al., 2004). *KITLG* gene is identified to be its pathogenic gene (Amyere et al., 2011). FPHH resembles the present cases in the generalized lentigo-like lesions on the body. However, the dyschromatosis also presented with symmetrical hypopigmented macules or patches and lacked other classical manifestations of FPHH, such as diffuse hyperpigmentation and café-au-lait macules. Histologically, FPHH has been reported to be characterized by an unchanged number of melanocytes and a Caucasian-like distribution pattern of melanosomes within keratinocytes in all lesions (Amyere et al., 2011; Zanardo et al., 2004). Alteration in melanocytes’ number participated in the *SASH1*-related dyschromatosis and the distribution pattern of melanosomes within keratinocytes is consistent with the previously reported distribution of melanosomes in keratinocytes from different skin types. These features distinguished our case from FPHH.

Other than the peculiar type of dyschromatosis (Zhou et al., 2013; Zhong et al., 2019; Wu et al., 2020; Liu et al., 2021; Cao et al., 2021; Yang et al., 2023), the *SASH1* mutations are also associated with another phenotype of skin pigmentation disorder: multiple lentigines (Shellman et al., 2015; Zhang et al., 2016; Wang et al., 2017; Araki et al., 2021; Kim et al., 2023). To date, a total of 22 *SASH1* mutations have been found associated with skin pigmentation changes (S587N was reported in the *Chinese Journal of Dermatology* and *Chinese Journal of Dermatovenereology*, S507F and c.49_54dupCCCCAGfanfa, c.1284 + 4A>G were reported in *Chinese Journal of Dermatovenereology*) (Table 1). Among them, mutations S519N, S516I, and S587N are associated with both multiple lentigines and the peculiar dyschromatosis phenotype (Table 1). The difference between these two phenotypes of pigmentary disorder with *SASH1* mutations relates to the presence or absence of hypopigmentation. Additionally, the scattered and mild hypopigmented spots and macules are also observed in *SASH1*-related multiple lentigines as reported by Shellman et al. (2015) and Zhang et al. (2016), respectively, thereby indicating that multiple lentigines might develop into the dyschromatosis under certain circumstance. In addition, both conditions were found related to the changes in the number of melanocytes in the epidermis: the number of melanocytes in normally and hyper-pigmented skin were similar but approximately twice than the normal controls in both multiple lentigines (Shellman et al., 2015) and dyschromatosis related with *SASH1* mutations. Hence, we conclude that these two conditions related to *SASH1* might be the same disease spectrum because of greater resemblance in their clinical phenotype, pathologic characteristics, and molecular genetics.

**TABLE 1:** *SASH1* mutations associated with pigmentation disorders.

Number	Clinical phenotypes	Inheritance pattern	Onset age	Nucleotide change	Amino acid change	References
1	Dyschromatosis	-	-	c.1525G>A	Glu509Lys	Zhou et al. (2013)
2		-	-	c.1544T>C	Leu515Pro	Zhou et al. (2013)
3		-/sporadic	-/1 year	c.1651T>G	Tyr551Asp	Zhou et al. (2013); the present study
4		AD	7 months	c.1651T>C	Tyr551His	Zhong et al. (2019)
5		AD	3 years	c.1784T>C	p.Met595Thr	Zhong et al. (2019)
6		AD	2 years	c.1553A>C	p.Gln518Pro	Wu et al. (2020)
7		AD	1 year	c.1547G>A	p.Ser516Asn	Liu et al. (2021)
8		AD	<1 year	c.1547G>T	p.Ser516Ile	Liu et al. (2021)
9		AD	4 years	c.1529G>A	p.Ser510Asn	Cao et al. (2021)
10		AD	1 year	c.1757T>C	p.Ile586Tyr	Yang et al. (2023)
11		AD	<1 year	c.1761C>G	p.Ser587Arg	Chinese journal of Dermatology
12		AD	1 year	c.1556G>A	p.Ser519Asn	the present study
13	Multiple lentigines	AD	2 years	c.1556 G>A	p.Ser519Asn	Shellman et al. (2015)
14		AD	3 years	c.1537 A>C	p.Ser513Arg	Zhang et al. (2016)
15		Sporadic	-/3 months	c.1525_1530dupAAGT	p.Leu511Lysfs*21	Zhang et al. (2016), Journal of Practical of Dermatology
16		AD	18 months	c.1519T>G	p.Ser507Ala	Wang et al. (2017)
17		Sporadic	8 months	c,1547G>T	p.Ser516Ile	Araki et al. (2021)
18		Sporadic	2 years	C,1574C>G	p.Thr525Arg	Araki et al. (2021)
19		AD	2 years	c.1592C>A	p.Ser531Tyr	Araki et al. (2021)
20		Sporadic	14 months	c.1758C>G	p.Ile586Met	Araki et al. (2021)
21		AD	4 years	c.1930C>T	p.Arg644Trp	Araki et al. (2021)
22		AD	8 months	c.1528A>T	p.Ser510Cys	Kim et al. (2023)
23		Sporadic	Since birth	c.1574C>T	P,Thr525Ile	Kim et al. (2023)
24		Sporadic	6 months	c.1520C>T	p.Ser507Phe	Journal of Practical Dermatology
25		Sporadic	6 months	c.1761 C>G	p.Ser587Arg	Journal of Practical Dermatology
26		Sporadic	3 months	c.1526_1527insAAGT	p.Leu511Lysfs*21	Journal of Practical Dermatology
27		AD	4 months	c.49_54dupCCCGAG	p.Pro17_Glu18dup	Chinese journal of Dermatology

Abbreviations: AD, autosomal dominant.

Overall, we reported a phenotype of dyschromatosis with *SASH1* mutations in a five-generation Chinese family and a sporadic case and examined the clinicopathologic features of this peculiar dyschromatosis. The clinical phenotypic, pathologic, and genetic differences between *SASH1*-related dyschromatosis and other dyschromatosis of autosomal dominant inheritance including DUH, DSH, or FPHH suggest that the *SASH1*-related dyschromatosis might be a distinct entity. Additionally, the high consistency in clinical manifestation, pathological expression, and genetic basis between the *SASH1*-related dyschromatosis and *SASH1*-related multiple lentigines suggests that these two conditions might be different phenotypes of a disease spectrum. Our findings not only help enhance a more comprehensive understanding of *SASH1*-related dyschromatosis but also provide a useful hint for the diagnosis of pigmentary dermatosis.

## Data Availability

The original contributions presented in the study are included in the article/Supplementary Material; further inquiries can be directed to the corresponding author.

## Supplementary material

SUPPLEMENTARY FIGURE S1 Premature canities of individual IV 9. Individual IV 9 was 30-year-old, and his hair had turned almost completely white. His hair was dyed, and the color of the proximal hair was white.

SUPPLEMENTARY FIGURE S2 Clinical features of an additional individual with similar manifestations. **(A)** Hypopigmented patches with hyperpigmented macules on his elbows. **(B)** Hypopigmented patches with hyperpigmented macules on the dorsa of his hand. **(C)** Diffuse hypopigmentation on his buttocks, and scattered macules of hyper- and hypopigmentation on his trunk. **(D)** Diffuse hypopigmentation with interspersed spots of hyperpigmentation on his lower limbs and some small islands of normally pigmented skin within the hypopigmentation.
