# Supplementary figures and images for: Case report: Clinicopathological characteristics of SASH1 mutation-related dyschromatosis: a rethinking of the classification of dyschromatosis
Source: Front Genet. 2025 Mar 6;16:1414129. doi: 10.3389/fgene.2025.1414129 (PMC11922891; doi:10.3389/fgene.2025.1414129)

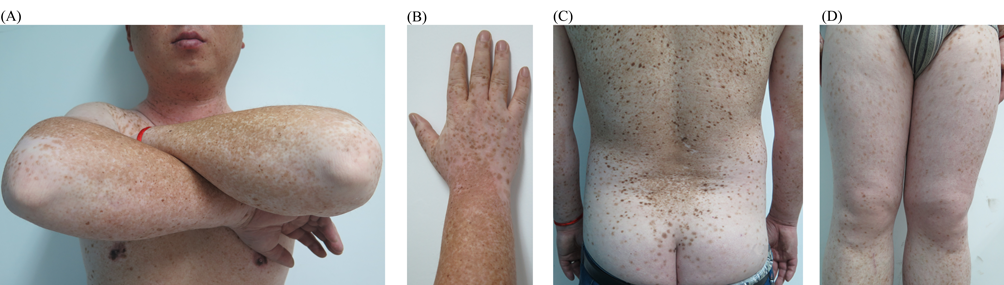

Supplement: Supplementary file 1 [file Image2.tif]

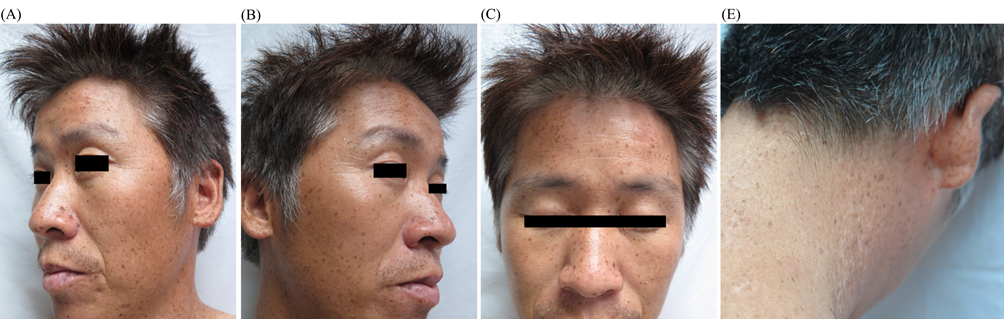

Supplement: Supplementary file 2 [file Image1.tif]
